# Trends and determinants of taking tetanus toxoid vaccine among women during last pregnancy in Bangladesh: Country representative survey from 2006 to 2019

**DOI:** 10.1371/journal.pone.0276417

**Published:** 2022-10-20

**Authors:** Md. Bony Amin, Nitai Roy, Amatul Elah Meem, Ekhtear Hossain, Md Aktarujjaman

**Affiliations:** 1 Faculty of Nutrition and Food Science, Patuakhali Science and Technology University, Patuakhali, Bangladesh; 2 Department of Biochemistry and Food Analysis, Patuakhali Science and Technology University, Patuakhali, Bangladesh; 3 Department of Biological Sciences and Chemistry, Southern University and A&M College, Baton Rouge, LA, United States of America; Hospital Femina, BRAZIL

## Abstract

**Background:**

Tetanus occurring during pregnancy is still an important cause of maternal and neonatal mortality in developing countries. This study estimated the trend of tetanus toxoid (TT) immunization coverage from 2006 to 2019 in Bangladesh, considering socio-demographic, socio-economic, and geospatial characteristics.

**Methods:**

The dataset used in this study was extracted from Multiple Indicator Cluster Surveys (2006, 2012–13, and 2019) including 28,734 women aged between 15–49 years. Data analysis was performed using cross-tabulation and logistic regression methods. Further, the spatial distribution of TT immunization coverage was also depicted.

**Results:**

The trend of TT immunization (81.8% in 2006 to 49.3% in 2019) and that of taking adequate doses of TT (67.1% in 2006 to 49.9% in 2019) has gradually decreased throughout the study period. Among the administrative districts, North and South-West regions had lower coverage, and South and West regions had relatively higher coverage of both TT immunization and that of adequate doses. Antenatal TT immunization (any dosage, inadequate or adequate) was significantly associated with lower age (AOR = 3.13, 1.55–6.34), higher education (AOR = 1.20, 1.03–1.40), living in urban areas (AOR = 1.17, 1.03–1.34), having immunization card (AOR = 5.19, 4.50–5.98), using government facilities for birth (AOR = 1.41, 1.06–1.88), and receiving antenatal care (ANC) (AOR = 1.51, 1.35–1.69). In addition, living in urban areas (AOR = 1.31, 1.10–1.55), having immunization cards (AOR = 1.62, 1.36–1.92), and choosing others’ homes for birth (AOR = 1.37, 1.07–1.74) were significantly associated with adequate TT immunization. However, higher education (AOR = 0.57, 0.44–0.74), having poor wealth index (AOR = 0.65, 0.50–0.83), and receiving ANC (AOR = 0.76, 0.63–0.92) had lower likelihood of taking adequate TT immunization.

**Conclusions:**

The gradual decline in the TT immunization rate in the present study suggests the presence of variable rates and unequal access to TT immunization, demanding more effective public health programs focusing on high-risk groups to ensure adequate TT immunization.

## Introduction

Tetanus is a deadly contagious infection caused by spore-forming bacteria *Clostridium tetani*. Though people of all ages can get tetanus, pregnant women are more susceptible to this fatal disease, especially those not being adequately immunized (at least two doses) with tetanus-toxoid-containing doses [1]. “Maternal Tetanus” during pregnancy or within 6 weeks of the end of pregnancy from wounds (birth, miscarriage, or abortion) causes serious consequences like lockjaw, muscle spasm as well as neonatal deaths https://www.ncbi.nlm.nih.gov/pmc/articles/PMC5496662/[1–5]. The tetanus-toxoid (TT) vaccine protects from such kinds of painful severe conditions that can lead to death [5].

Tetanus is highly responsible for neonatal deaths every year. Based on statistics from 2002, it is estimated that about 180,000 neonates died, which represents 5% of all neonatal deaths, among 218,000 cases in India due to inadequate TT immunization [6]. Besides each year about 15000 to 30000 maternal deaths occur for the lack of sufficient TT doses. Comparatively, maternal tetanus didn’t get much attention while neonatal tetanus got the core focus [7, 8]. Studies found that at least two doses of TT vaccine are required to build protection against maternal and neonatal tetanus in childbearing or pregnant women. Only the first dose does not have significant protective value or too little contribution against this fatal disease [9–11]. Tetanus vaccine develops safeguard by elevating protective antibody levels in more than 80% of receivers after 2 doses [12]. Besides, Two or more TT doses during pregnancy offer passive fetus immunity and have been revealed to lessen neonatal tetanus mortality by 96% [13–15].

Notably, there are 59 high-risk countries for the prevalence of maternal and neonatal tetanus [16]. But much advancement has been seen in the past two decades in taking tetanus toxoid vaccines to prevent risks and deaths. In 2017, for example, WHO estimated that 30,848 newborns died from neonatal tetanus worldwide, which represented an 85% reduction from the situation in 2000 [17]. Women worldwide are taking sufficient doses of the TT vaccine. The total prevalence of getting TT immunization during women’s last pregnancy was 96.3% and that of receiving at least two doses was 82.12% in Sierra Leone [13]. The prevalence of women who took adequate tetanus vaccination was 60.0% in Sudan [18]. Another study of Gambia showed the overall prevalence of TT uptake in women was 88.2% whereas only 34.8% took adequate doses [19]. These studies have identified that age, education, ethnicity, antenatal care (ANC) visit, parity, use of newspaper and radio, and wealth index were linked with TT vaccination coverage [13, 18, 19].

Despite progress, it is still a burning issue for developing countries greatly in Asia and Africa [20, 21]. Studies found some primary reasons behind this problem: poor vaccine access and lack of knowledge of sufficient vaccination coverage [22]. In Bangladesh, the percentage of Tetanus-Toxoid immunization during pregnancy extended from 5% to 67% from 1985-to 1995 [23]. An earlier study from Bangladesh reported that 89% of women having a child under 1 year of age and 52% of reproductive age took at least one TT immunization. Furthermore, 85% of mothers with children under one year old, 64% of married women who had previously been pregnant, and 47% of women of reproductive age reported receiving at least two doses of TT vaccination [24]. Another study depicts that only 22.3% of pregnant women took one TT and 56.3% had more than 2 TT immunizations [25]. In addition, Abir et al. from Bangladesh reported that 26.0% of mothers had taken one TT dose, and 55.6% had received two or more doses [26]. These studies did not investigate the trends of TT coverage over the years, which is the focus of the present study.

Notably, after India, China, and Pakistan; Bangladesh was identified as the fourth-highest country for neonatal tetanus with estimated cases of 41000 annually [23]. Vulnerability in pregnant women mostly resides in the rural part of our country where TT is a continuing program under EPI regarding maternal and neonatal tetanus elimination. Bangladesh government has taken the initiative of the EPI program since 7th April 1979 [27]. However, to our knowledge, changes over time in TT vaccination coverage have not been investigated. Little is known about the determinants of TT vaccination coverage in Bangladesh, a country being a hotspot for infectious diseases. Better understanding the determinants of TT vaccination coverage may also assist in tailoring program interventions. Therefore, it is important to understand the general trend of TT vaccination coverage, and the issues surrounding vaccination. This study aims to demonstrate the trend and determinants of TT immunization among Bangladeshi pregnant women using data from the United Nations Children’s Fund (UNICEF) from 2006 to 2019.

## Methodology

### Data overview

This cross-sectional study used survey data sets from 2006, 2012–13, and 2019 of the Bangladesh Multiple indicator cluster surveys (MICS), an international survey initiative carried out by the Bangladesh Bureau of Statistics in collaboration with UNICEF. The MICS were developed and supported by UNICEF “focusing mainly on issues that directly affect the lives of children and women” allowing countries to generate evidence and recommended strategies to monitor the progress toward millennium development goals [28]. The survey (2019) employed a two-stage stratified cluster sampling method where each of the 64 districts was considered as the sampling strata. The primary sampling units were enumeration areas (EAs) based on the 2011 Bangladesh population, with housing census and households serving as secondary sampling units. Using the probability proportional to size (PPS) method, a total of 3220 EAs were selected from all 64 strata in the first step of sampling. In the second stage, a random systematic selection was utilized to choose a sample of 20 households from each sampled EA [29]. For the 2012–13 round, a two-stage stratified cluster sampling approach was used to determine the survey samples. Administrative districts were designated as priority districts and non-UNDAF districts under the United Nations Development Assistance Framework (UNDAF). 50 sample clusters were chosen from each of the 20 UNDAF districts, and 40 sample clusters were chosen from each of the 44 non-UNDAF districts. A systematic random selection technique was used to choose sample households in each cluster from a list of households [30]. In round 2006, a multi-stage, stratified cluster sampling approach was used for the selection of the survey sample. In each enumeration region, households were sequentially numbered from 1 to 100 (or more), and 35 households were selected using systematic selection procedures [31]. We extracted data for women aged 15–49 years from the dataset of three rounds of the survey. The total number of households surveyed from 2006 to 2019 was 206568. In the raw data, the total number of women aged 15–49 years was 186028 (unweighted). After cleaning the missing values, the remaining 11812, 7783, and 9139 (weighted) women from 2006, 2012–13, and 2019, respectively were included in the final analysis.

### Outcome variables

The two outcome variables used for this study were similar to previous studies [13, 18, 19]. The question that was asked to the participants was whether or not they took TT vaccination during their last pregnancy. In addition, based on WHO recommendations, receiving at least two doses of TT is considered adequate while less than 2 doses are regarded as inadequate [19].

### Finding covariates

We considered explanatory variables according to their know association with the uptake of TT immunization [25, 32–34]. The variables included age (15–19, 20–24, 25–29, 30–34, 35–39, 40–44, 45–49 years), education (never attended school, primary incomplete, primary complete, secondary incomplete, secondary completed or higher), area (rural, urban, tribal), wealth status (poorest, second, middle, fourth, Richest), administrative region (Sylhet, Barishal, Chittagong, Dhaka, Khulna, Mymensingh, Rajshahi, Rangpur), ANC care (yes, no, other), immunization card (no, card seen, card not seen, other), and place of delivery (private service, government service, respondents home, other). We excluded social media exposure variables like watching television, using computers, and listening to the radio because it was not available in all three datasets.

### Statistical analyses

IBM SPSS statistics 26.0 version was used for the analysis. First, simple descriptive tests were done to observe the exact group frequencies, percentages, minimum, maximum, range, etc. Then, Pearson Chi-square tests were carried out to imprint the association of covariates with the two dependent variables. Multicollinearity between independent variables was measured using correlation coefficient and the value of all the variables was less than 0.5 indicating the absence of multicollinearity. After that, logistic regression models for the binary outcome were used to analyze the multivariable association between covariates and outcomes. Tableau Desktop version 2021.2 was used to create the line chart and ArcGIS v10.5 to visualize the % changes occurring across 64 districts of Bangladesh. All tests were two-sided and had statistically eligible significant values below 0.05 with 95% confidence intervals. Forest plots were used for the graphical representation of the significant findings.

### Ethical clearance

This study analyzed survey data from UNICEF, where all the personally identifiable information of participants had been removed. The national statistical office, Bangladesh Bureau of Statistics, and UNICEF obtained informed consent from survey participants before their participation. Because we used publicly available de-identified data, our work was exempt from full ethical review process and approved by the Research and Ethical Committee of Department of the Biochemistry and Food Analysis, Patuakhali Science and Technology University (approval no.: BFA 12/01/2022:03). In addition, upon completing the registration process, the authors were granted permission to download and use the datasets. The data are available online: http://mics.unicef.org/surveys.

## Results

### Changes in the prevalence of taking tetanus toxoid in Bangladesh

**Fig 1:**
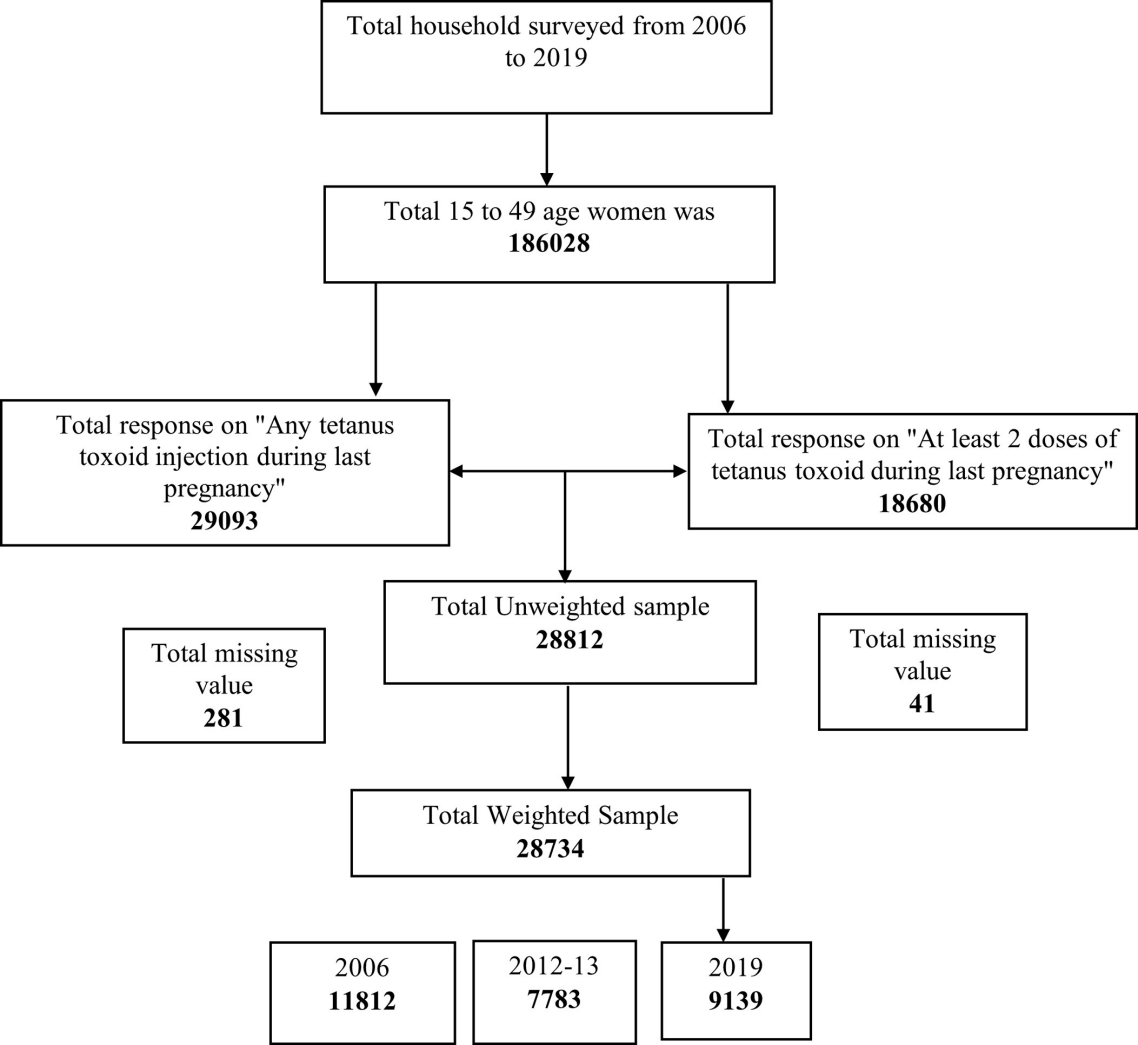
Study profile.

**Fig 2:**
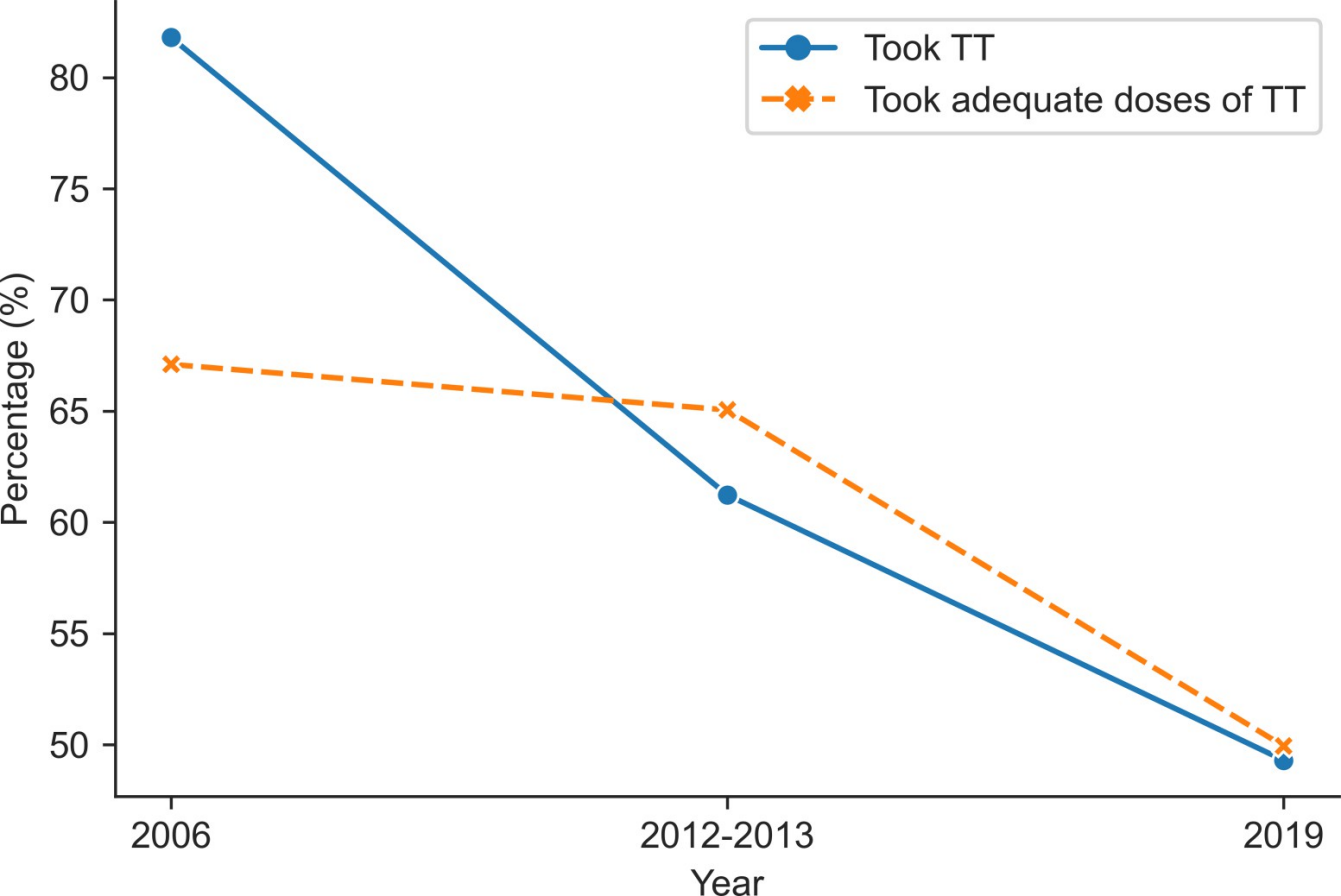
Changes in the prevalence of taking tetanus toxoid among pregnant women aged 15–49 years in Bangladesh (2006–2019).

After excluding the missing values, 28,734 pregnant women aged 15–49 years were included in our study (Fig 1). As shown in Fig 2, there was a decreasing trend in the percentage of taking TT immunization among pregnant women aged 15–49 years. The percentage of taking TT injections steadily decreased from 81.8% in 2006 to 49.3% in 2019. Similarly, the percentage of taking adequate doses of TT injection also steadily decreased from 67.1% in 2006 to 49.9% in 2019.

### Prevalence of taking tetanus toxoid in Bangladesh

Table 1 shows that there have been prominent changes in TT vaccination coverage in Bangladesh over the last fourteen years. The prevalence of taking TT vaccination among pregnant women aged 15–49 years decreased consistently from 2006 to 2019 for age, education, area, administrative region (division), immunization card, and ANC visit. Similarly, by wealth index and place of delivery, TT vaccination coverage consistently decreased from 2006 to 2019, except for the 2019 round. Although, the prevalence was slightly raised for delivery at the respondent’s homes for the 2019 round. The prevalence of taking adequate TT vaccination decreased consistently from 2006 to 2019 for age, administrative region (division), and ANC visit. Consequently, the trend of taking adequate TT declined from 2006 to 2019, except for the 2006 round for areas, the 2019 round for wealth index and immunization card, and the 2012–13 round for place of delivery. In addition, the prevalence of adequate TT vaccination in all education categories was reduced from 2006 to 2019, although for the 2006 and 2012–13 rounds the result was not significant.

**Table 1:** Prevalence of taking tetanus toxoid among pregnant women aged 15–49 years in Bangladesh (2006–2019).

Variables & Categories	Took TT						Doses of TT					
	2006		2012–13		2019		2006		2012–13		2019	
	Total (%)	Yes (%)	Total (%)	Yes (%)	Total (%)	YES (%)	Total (%)	Adequate (%)	Total (%)	Adequate (%)	Total (%)	Adequate (%)
**Age**												
15–19	2353 (19.9)	2064 (87.7)	911 (11.7)	670 (73.5)	1241 (13.6)	866 (69.8)	2064 (21.4)	1577 (76.4)	670 (14.0)	436 (65.1)	866 (19.2)	492 (56.8)
20–24	4087 (34.6)	3474 (85.0)	2723 (35.0)	1715 (63.0)	2932 (32.1)	1524 (52. 0)	3474 (35.9)	2365 (68.1)	1715 (35.9)	1195 (69.7)	1524 (33.8)	805 (52.8)
25–29	2925 (24.8)	2377 (81.3)	2307 (29.6)	1371 (59.4)	2513 (27.5)	1103 (43.9)	2377 (24.6)	1477 (62.1)	1371 (28.7)	877 (64.0)	1103 (24.5)	503 (45.6)
30–34	1536 (13.0)	1128 (73.4)	1080 (13.9)	605 (56.0)	1604 (17.6)	653 (40.7)	1128 (11.7)	672 (59.6)	604 (12.7)	344 (57.0)	654 (14.5)	294 (45.0)
35–39	726 (6.1)	505 (69.6)	548 (7.0)	310 (56.6)	678 (7.4)	294 (43.4)	505 (5.2)	316 (62.6)	310 (6.5)	189 (61.08)	295 (6.5)	124 (42.2)
40–44	147 (1.2)	93 (63.3)	174 (2.2)	87 (50.0)	133 (1.5)	47 (35.3)	93 (1.0)	60 (64.5)	87 (1.8)	54 (62.1)	47 (1.0)	20 (42.6)
45–49	38 (0.3)	26 (68.4)	40 (0.5)	14 (35.0)	36 (0.4)	17 (47.2)	26 (0.3)	21 (80.8)	14 (0.3)	10 (71.4)	17 (0.4)	11 (64.7)
**X** ^**2**^	268.21***		103.15***		313.46***		145.11***		37.16***		45.82***	
**Education**												
Primary incomplete	1888 (16.0)	1519 (80.5)	1021 (13.1)	629 (61.6)	13 (0.1)	6 (46.2)	1519 (15.7)	1057 (69.6)	629 (13.2)	409 (65.0)	6 (0.1)	2 (33.3)
Primary completed	1540 (13.0)	1281 (83.2)	1220 (15.7)	736 (60.3)	2124 (23.2)	1041 (49.0)	1281 (13.3)	866 (67.6)	736 (15.4)	488 (66.3)	1041 (23.1)	547 (52.5)
Secondary incomplete	3401 (28.8)	2959 (87.0)	2970 (38.2)	1886 (63.5)	2299 (25.2)	1237 (53.8)	2959 (30.6)	1961 (66.3)	1886 (39.5)	1231 (65.3)	1237 (27.5)	627 (50.7)
Secondary completed or higher	1248 (10.6)	1083 (86.8)	1140 (14.6)	740 (64.9)	3877 (42.4)	1870 (48.2)	1083 (11.2)	699 (64.5)	740 (15.5)	456 (61.6)	1869 (41.5)	884 (47.3)
Non-standard curriculum^**a**^	38 (0.3)	32 (84.2)	-	-	-	-	32 (0.3)	25 (78.1)	-	-	-	-
Never attend school	3696 (31.3)	2793 (75.6)	1431 (18.4)	781 (54.6)	826 (9.0)	351 (42.5)	2793 (28.9)	1880 (67.3)	781 (16.4)	522 (66.8)	351 (7.8)	188 (53.6)
**X** ^**2**^	183.83***		40.14***		35.87***		10.34		5.47		10.82*	
**Area**												
Urban	3005 (25.4)	2512 (83.6)	1644 (21.1)	1102 (67.0)	2006 (21.9)	948 (47.3)	2512 (26.0)	1655 (65.9)	1101 (23.1)	761 (69.1)	948 (21.0)	516 (54.4)
Tribal^**b**^	100 (0.8)	72 (72.0)	-	-	-	-	72 (0.7)	54 (75.0)	-	-	-	-
Rural	8707 (73.7)	7084 (81.4)	6137 (78.9)	3669 (59.8)	7134 (78.1)	3557 (49.9)	7084 (73.3)	4779 (67.5)	3670 (76.9)	2344 (63.9)	3557 (79.0)	1733 (48.7)
**X** ^**2**^	14.09**		28.71***		4.24*		4.14		10.27**		9.76**	
**Wealth index quintiles**												
Poorest	2883 (24.4)	2253 (78.1)	1781 (22.9)	1022 (57.4)	1943 (21.3)	970 (49.9)	2253 (23.3)	1537 (68.2)	1022 (21.4)	656 (64.2)	970 (21.5)	493 (50.8)
Second	2513 (21.3)	2036 (81.0)	1582 (20.3)	965 (60.0)	1721 (18.8)	861 (50.0)	2036 (21.1)	1406 (69.1)	965 (20.2)	605 (62.7)	861 (19.1)	399 (46.3)
Middle	2221 (18.8)	1812 (81.6)	1494 (19.2)	901 (60.3)	1742 (19.1)	871 (50.0)	1812 (18.7)	1243 (68.6)	901 (18.9)	582 (64.6)	870 (19.3)	442 (50.8)
Fourth	2226 (18.8)	1865 (83.8)	1378 (17.7)	871 (63.2)	1805 (19.8)	873 (48.4)	1865 (19.3)	1184 (63.5)	871 (18.3)	548 (62.9)	873 (19.4)	451 (51.7)
Richest	1969 (16.7)	1702 (86.4)	1548 (19.9)	1013 (65.4)	1927 (21.1)	930 (48.3)	1702 (17.6)	1118 (65.7)	1012 (21.2)	714 (70.6)	930 (20.6)	463 (49.8)
**X** ^**2**^	61.39***		25.49***		2.47		19.23**		18.00**		6.07	
**Division**												
Barisal	732 (6.2)	634 (86.6)	465 (6.0)	329 (70.8)	507 (5.5)	323 (63.7)	635 (6.6)	464 (73.1)	329 (6.9)	239 (72.6)	322 (7.1)	190 (59.0)
Chittagong	2536 (21.5)	2160 (85.2)	1769 (22.7)	1236 (69.9)	1982 (21.7)	1120 (56.5)	2160 (22.3)	1513 (70.0)	1236 (25.9)	912 (73.8)	1120 (24.9)	644 (57.5)
Dhaka	3667 (31.0)	2971 (81.0)	2470 (31.7)	1480 (59.9)	2192 (24.0)	994 (45.3)	2972 (30.7)	2001 (67.3)	1479 (31.0)	997 (67.4)	994 (22.1)	531 (53.4)
Khulna	1141 (9.7)	918 (80.5)	753 (9.7)	433 (57.5)	927 (10.1)	456 (49.2)	918 (9.5)	529 (57.6)	433 (9.1)	182 (42.0)	456 (10.1)	172 (37.7)
Mymensingh^**c**^	-	-	-	-	702 (7.7)	390 (55.6)	-	-	-	-	391 (8.7)	193 (49.4)
Rajshahi	2716 (23.0)	2213 (81.5)	839 (10.8)	500 (59.6)	1069 (11.7)	549 (51.4)	2213 (22.9)	1474 (66.6)	499 (10.5)	313 (62.7)	549 (12.2)	203 (37.0)
Rangpur^**d**^	-	-	871 (11.2)	557 (63.9)	995 (10.9)	455 (45.7)	-	-	557 (11.7)	323 (58.0)	455 (10.1)	202 (44.4)
Sylhet	1019 (8.6)	771 (75.7)	617 (7.9)	237 (38.4)	765 (8.4)	218 (28.5)	771 (8.0)	508 (65.9)	237 (5.0)	139 (58.6)	218 (4.8)	114 (52.3)
**X** ^**2**^	59.73***		218.71***		247.36***		56.92***		172.14***		111.28***	
**Has immunization card**												
Yes (card seen)	3477 (29.4)	3173 (91.3)	2157 (27.7)	1605 (74.4)	1916 (21.0)	1195 (62.4)	3174 (32.8)	2063 (65.0)	1605 (33.7)	1138 (70.9)	1195 (26.5)	623 (52.1)
Yes (card not seen)	4857 (41.1)	4236 (87.2)	3047 (39.2)	1936 (63.5)	2271 (24.9)	1220 (53.7)	4235 (43.8)	2920 (68.9)	1936 (40.6)	1179 (60.9)	1221 (27.1)	579 (47.4)
No	3476 (29.4)	2258 (65.0)	2570 (33.1)	1227 (47.7)	4950 (54.2)	2089 (42.2)	2258 (23.4)	1505 (66.7)	1228 (25.7)	787 (64.1)	2089 (46.4)	1047 (50.1)
**X** ^**2**^	968.88***		361.89***		248.47***		13.13**		39.38***		5.43	
**Place of delivery**												
Respondent’s home	7617 (64.8)	6065 (79.6)	4875 (62.7)	2971 (60.9)	3549 (38.8)	1695 (47.8)	6065 (63.0)	4008 (66.1)	2971 (62.3)	1963 (66.1)	1695 (37.6)	893 (52.7)
Government services^**e**^	881 (7.5)	778 (88.3)	1032 (13.3)	664 (64.3)	1455 (15.9)	724 (49.8)	778 (8.1)	511 (65.7)	665 (13.9)	416 (62.6)	724 (16.1)	358 (49.4)
Other ^**f**^	2245 (19.1)	1908 (85.0)	465 (6.0)	255 (54.8)	714 (7.8)	368 (51.5)	1908 (19.8)	1373 (72.0)	254 (5.3)	150 (59.1)	368 (8.2)	203 (55.2)
Private services^**g**^	1015 (8.6)	872 (85.9)	1405 (18.1)	881 (62.7)	3421 (37.4)	1717 (50.2)	872 (9.1)	560 (64.2)	881 (18.5)	576 (65.4)	1718 (38.1)	795 (46.3)
**X** ^**2**^	76.27***		13.64**		6.01		27.20***		7.24		18.42***	
**Received ANC**												
Yes	6617 (56.1)	5682 (85.9)	5241 (67.4)	3376 (64.4)	7568 (82.8)	3872 (51.2)	5682 (58.9)	3717 (65.4)	3377 (70.8)	2156 (63.8)	3871 (85.9)	1894 (48.9)
No	5169 (43.9)	3961 (76.6)	2540 (32.6)	1395 (54.9)	1572 (17.2)	633 (40.3)	3960 (41.1)	2751 (69.5)	1395 (29.2)	949 (68.0)	633 (14.1)	354 (55.9)
**X** ^**2**^	166.54***		65.01***		61.82***		17.36***		7.61**		11.33**	
Total	11812 (100)	9667 (81.8)	7783 (100)	4772 (61.3)	9139 (100)	4504 (49.3)	9667 (100)	6488 (67.1)	4771 (100)	3105 (65.1)	4505 (100)	2249 (49.9)

*p<0.05

**p<0.01

***p<0.001 (p indicates *P*-Value)

a = “Non-standard curriculum” data were not collected in 2012–13 5 and 2019.

b = “Tribal” data were not collected in 2012–13 and 2019.

c = Mymensingh division was established in 2015.

d = Rangpur division was established in 1 July 2010.

e = Government services include “Government hospital, Govt. clinic, Govt. health center and Other public services”

f = “Other” include “Other home and other values provided by MICS.

g = Private services include “Private clinic, private hospital, private maternity home and other private medical”

### Spatial distribution of change rate in tetanus toxoid vaccination status from 2006 to 2019

**Fig 3:**
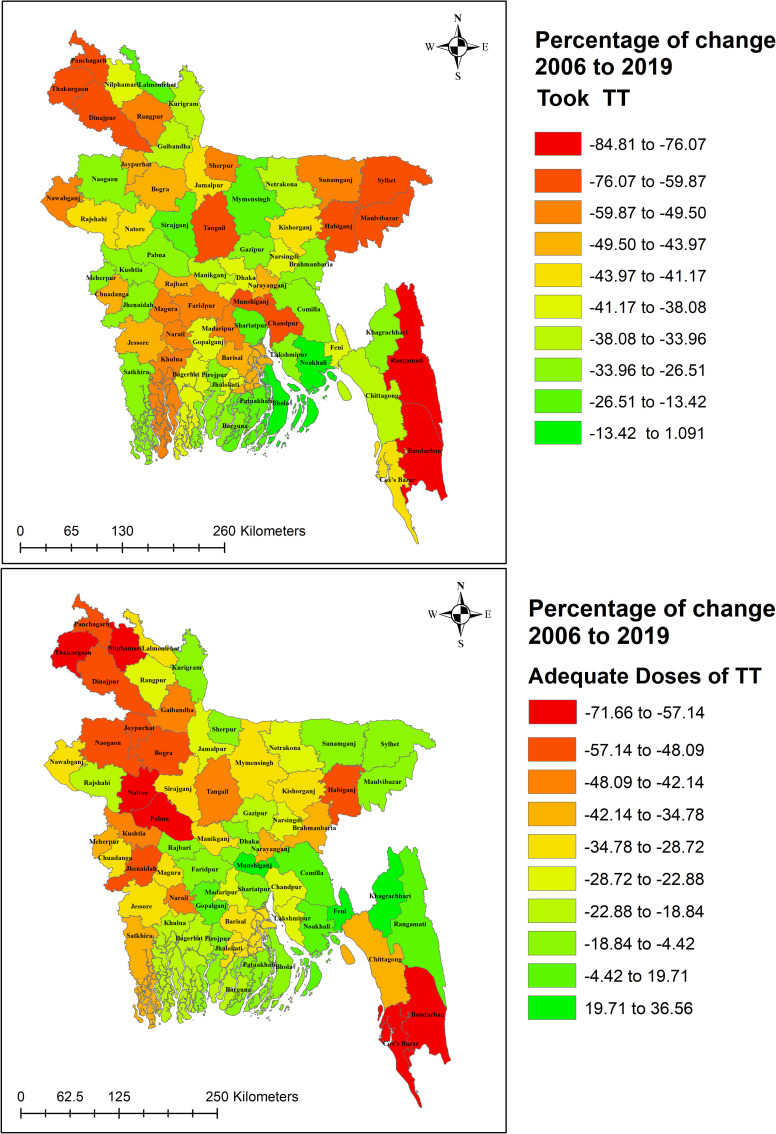
Spatial distribution of change rate of tetanus toxoid vaccination status from 2006 to 2019. Republished from [35] under a CC BY license, with permission from [Bangladesh Agricultural Research Council], original copyright [2014].

Fig 3 and S1 Table represents the geographical pattern of the change rate in TT immunization coverage from 2006 to 2019. The majority of regions showed a cutback in TT uptake over time from 2006 to 2019, while some areas showed a slightly positive change rate (-13.42 to 1.091). Similarly, from 2006 to 2019 all areas experienced a reduction in adequate TT uptake except for some of the areas having positive changes (19.71 to 36.56).

### Determinants of taking tetanus toxoid vaccine in Bangladesh

**Fig 4:**
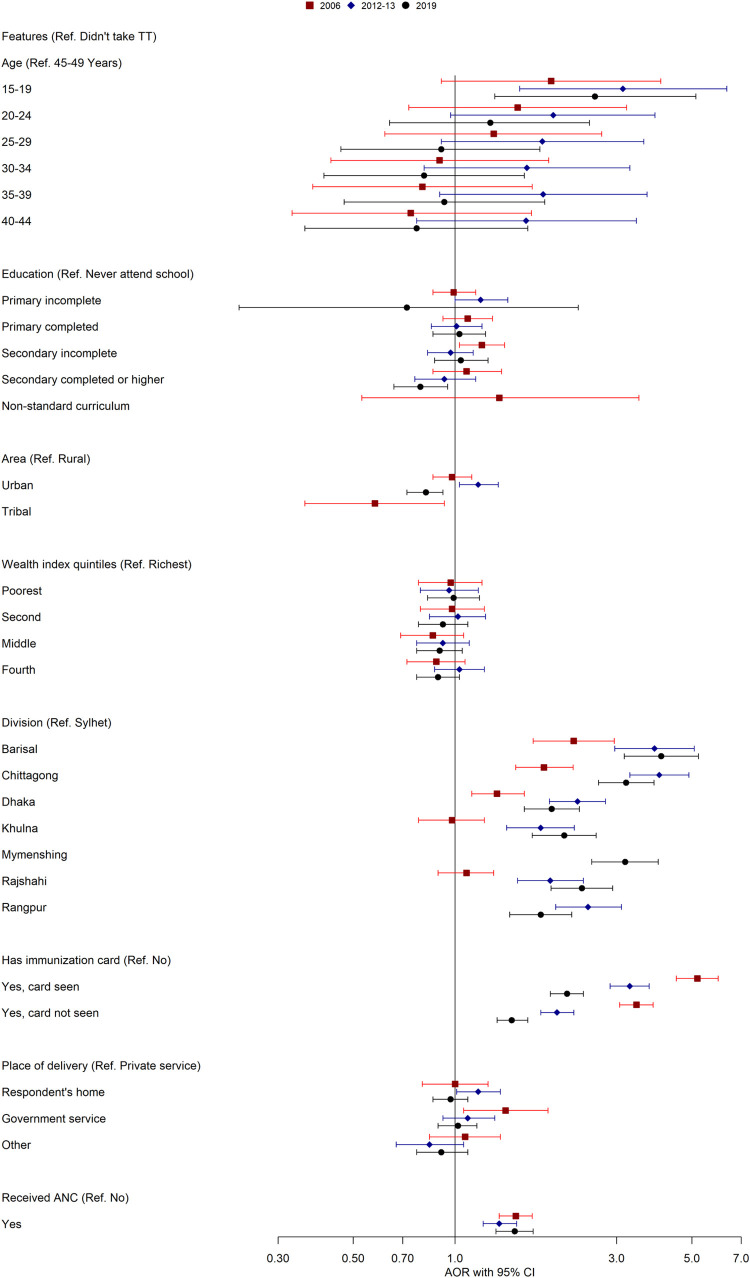
Determinants of taking tetanus toxoid vaccine in Bangladesh (2006–2019).

Fig 4, S2 and S3 Tables show the factors associated with the TT immunization coverage among women aged 15–49 years in terms of CORs and AORs in Bangladesh. Women aged 15 to 19 had a significantly higher uptake of TT vaccine than women aged 45 to 49 in 2012–13 (AOR = 3.13, 1.55–6.34), and 2019 (AOR = 2.59, 1.31–5.14). The findings also revealed that education was significantly associated with TT immunization. It was observed that the odds of having TT immunization were increased as the educational level increased, and that of women with incomplete secondary school (AOR = 1.20, 1.03–1.40) and incomplete primary school (AOR = 1.19, 1.00–1.43) were more likely to be vaccinated compared to uneducated women for 2006 and 2012–13. However, women with secondary or higher education were less likely (AOR = 0.79, 0.66–0.95) to receive the TT vaccine for 2019. In addition, the study found that women from the urban area took significantly higher TT than rural counterparts (AOR = 1.17, 1.03–1.34) from 2012-to 13. In contrast, women living in urban and tribal areas were 18% and 32% less likely to have TT immunization in 2019 and 2006. The likelihood of being TT immunized was significantly lower in the Sylhet division than in all other administrative regions. Remarkably, Barishal and Chittagong divisions had higher odds ratios compared to other divisions across all three survey years. Women who had immunization cards and were able to show that or unable to show had significantly higher TT immunization coverage in all survey years. Women who chose government service for delivery took significantly higher TT vaccine (AOR = 1.41, 1.06–1.88) than those who chose private service for delivery in 2006, although other survey years remained insignificant. Those who received antenatal care had significantly higher TT uptake (AOR = 1.51, 1.35–1.69), (AOR = 1.35, 1.21–1.52), and (AOR = 1.50, 1.32–1.70) in 2006, 2012–13, and 2019, respectively than who did not receive antenatal care.

### Determinants of taking adequate tetanus toxoid vaccine in Bangladesh

**Fig 5:**
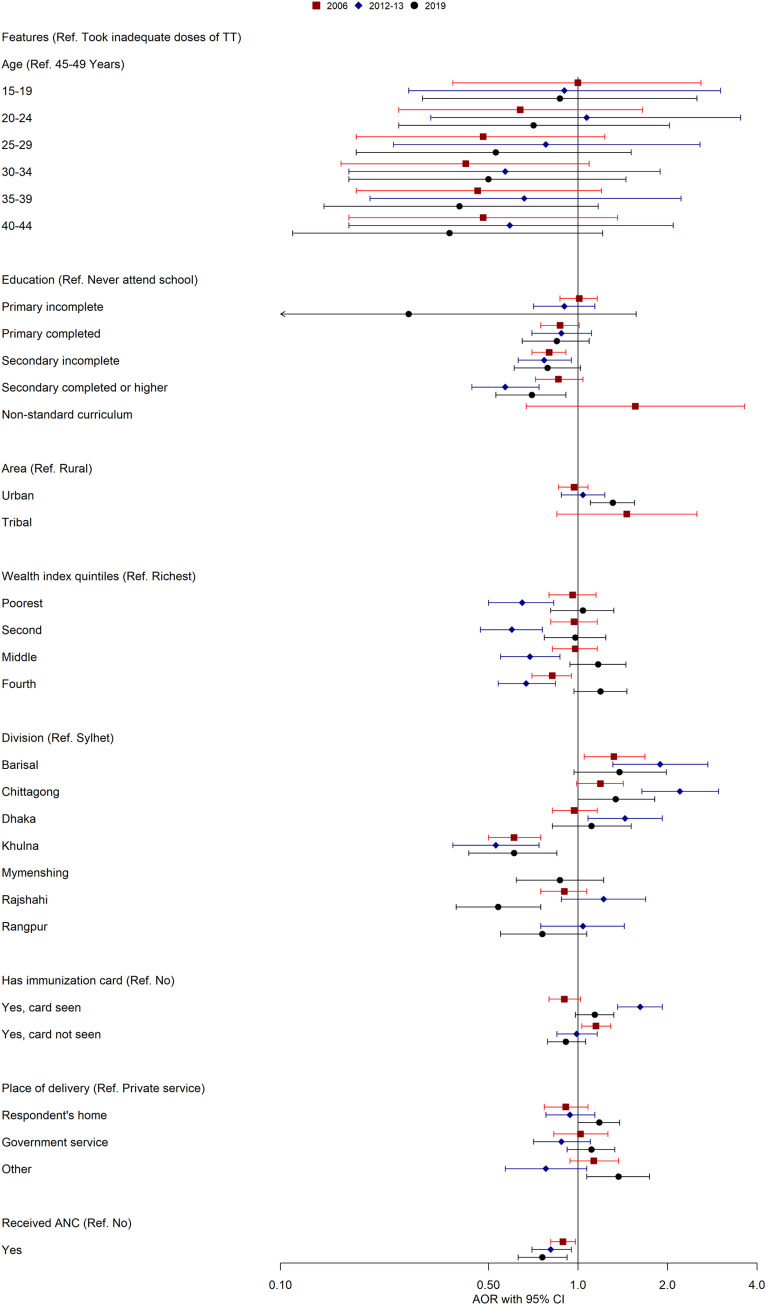
Determinants of taking adequate tetanus toxoid vaccine in Bangladesh (2006–2019).

The education of the participant is a significant covariate of adequate TT immunization. This data demonstrates that in 2006 and 2012–13, the women who did not complete their secondary education had significantly lower (AOR = 0.80, 0.70–0.91) and (AOR = 0.77, 0.63–0.95) uptake of adequate TT doses than uneducated women (Fig 5, S2 and S4 Tables). Similarly, the women who completed secondary or higher education had significantly lower (AOR = 0.57, 0.44–0.74) and (AOR = 0.70, 0.54–0.91) TT immunization in the 2012–13 and 2019 survey years than those who never attended school. In addition, the study results disclosed that the odds of utilizing adequate TT immunization were significantly higher in urban areas than in their rural counterparts (AOR = 1.31, 1.10–1.55) in 2019. We also found that having a lower wealth quintile was significantly associated with lower odds of receiving adequate TT immunization in 2006 and 2012–13. In comparison to the Sylhet division, women living in Dhaka, Barishal, and Chittagong showed higher odds of adequate TT immunization for 2006 and 2012–13 survey years. Conversely, the likelihood of being adequate TT immunization was significantly lower in the Khulna division than Sylhet division across all survey years. In 2012–13, women who had immunization cards and were able to show that had significantly higher (AOR = 1.62, 1.36–1.92) TT immunization coverage than women who had no immunization cards. Similarly, women who had immunization cards and were unable to show that had significantly higher (AOR = 1.15, 1.03–1.29) TT immunization coverage. Interestingly, women who chose their own homes and others’ homes for delivery had significantly higher odds of TT immunization (AOR 1.18) and (AOR 1.37) than those who chose private services for delivery in 2019. Women who received ANC were significantly less likely (AOR = 0.89, 0.81–0.98, AOR = 0.81, 0.70–0.95), and (AOR = 0.76, 0.63–0.92, respectively) to take adequate TT doses than women who did not receive ANC during pregnancy across all survey years.

## Discussion

This study investigated the factors associated with TT immunization coverage among pregnant women aged 15–49 years. We observed that for MICS (2006), MICS (2012–13) and MICS (2019) the prevalence of TT immunization was 81.8%, 61.3%, and 49.3%, respectively. Moreover, the prevalence of adequate TT doses was 67.1%, 65.1%, and 49.9% for MICS (2006), MICS (2012–13), and MICS (2019), respectively. Overall, the prevalence shows a decreasing trend across the survey years in Bangladesh. The prevalence of adequate TT doses was 49.9% from the latest survey (MICS 2019), which is low as compared to the study [36] showed that sufficient TT immunization among pregnant women was 75% worldwide, 95% in South East Asia, 63% in Africa, and 53% in East Mediterranean.

The high socio-demographic condition is associated with TT immunization. The higher TT immunization rate among younger women in the present study might be attributed to the improved formal female education and access to modern media outlets. By previous studies, age is an insignificant factor regarding adequate doses of TT immunization [13]. In contrast, the association between age and adequate doses of TT immunization has been demonstrated in other countries [18, 19]. However, we determined that age is a significant factor regarding any doses of TT immunization only for 2012–13 and 2019. Further studies are warranted to comprehend this connection properly. With some exceptions, this study suggests that increasing the level of education increases the uptake of TT immunization and adequate doses of TT immunization, in line with other studies [37, 38]. Because educated women might be more likely to have decision-making power regarding their health and education may improve the level of knowledge about the deleterious effects of tetanus [18]. In our study, urban women were more likely to take sufficient TT immunization, in agreement with other studies [39, 40], which might reflect the improved access to healthcare facilities in the urban area. Therefore, interventions especially targeting uneducated rural women are needed to improve the current scenario of TT immunization in Bangladesh. However, another study in Afghanistan concluded that urban women had lower odds of being sufficiently vaccinated, and may be offered less TT immunization than their rural counterparts due to less knowledgeable ANC providers and less vaccine availability [41].

Bangladesh has achieved remarkable health improvements during the last two decades [42]. More recently, Bangladesh was commended as an example of “good health at low cost” [42]. However, socioeconomic inequality in health, especially in maternal and child mortality remains a disturbing reality in Bangladesh [42]. Similar to our findings, studies revealed that increasing the wealth index of women in the household is protective against tetanus compared to a poor wealth index [18]. The financial reason might contribute to women from wealthy households getting easier access to healthcare facilities compared to those from poor households as well as women from low economic status are challenged with availability and high maternity and transportation cost when seeking health care. Moreover, women from the poor wealth index might be engaged in other activities to fulfill their basic needs, limiting their time to utilize healthcare services compared with the richest suggesting that strategies such as improvement of health literacy and logistic support could be taken to enable coverage and equity of TT immunization across women on areas with poor wealth index. However, this finding opposes a study conducted in the Gambia that showed that the wealth quintile did not affect TT immunization [19].

This study showed that women who uptake TT immunizations or adequate TT doses using immunization cards had a higher chance of being protected from tetanus. This finding is similar to studies conducted in Ethiopia and Ghana [22, 43], which encouraged women to promote immunization card retention as well as other records of health facilities as a mechanism to improve the immunization rate. The present study demonstrated that birth at government facilities and home birth were protective factors for TT immunization and adequate TT doses. The presence of user fees for maternal health services and immunization might appear to be a major barrier to increasing TT immunization in private clinics suggesting exemption of user fees for maternal and child immunization [44, 45]. However, the current study contradicts the findings of an earlier study [46] that showed that place of delivery did not affect TT immunization.

Another important factor significantly associated with TT immunization was ANC follow-up, consistent with previous studies conducted in different countries worldwide [13, 18]. This might be because higher ANC follow-up increased awareness of the importance of TT immunization and ANC visits also offer interventions and provides critical healthcare functions that might be crucial to health and well-being. Interestingly, women who attend high ANC visits are less likely to receive adequate TT immunization. This suggests that the burden of user fees and transportation costs for ANC might serve as a barrier to care and further discourage the continuation of TT immunization, contributing to the evidence that user fees exemption policies may reduce the inequities in access to care [47].

The spatial analysis discovered some district-wise variations in the change rate of TT immunization from 2006 to 2019, with Rangamati and Bandarban districts experiencing the worst. Moreover, regarding adequate TT doses, the situation was worse in Bandarban, Cox’s Bazar, Pabna, Natore, Thakurgaon, and Nilphamari districts. Therefore, the policy focus here would be on the peripheral districts and the hill tracts in Bangladesh to improve women’s health literacy in remote areas and ensure maternal healthcare access.

This study has several strengths. First, this is the first study to demonstrate trends of TT immunization among women during the last pregnancy in Bangladesh. Second, we used data from three nationally representative datasets and the findings can be generalized to a whole nation. However, this study does have some limitations. Due to the cross-sectional nature of the study, causal inference of the association between TT immunization and women’s health cannot be drawn. Since this study asks participants about past exposure and numerous vaccines indicated in the period in question, recall bias may occur. Social media exposure variables like watching television, using a computer, and listening to radiofrequency were not common in the three datasets and are missing from the analysis. Secondary data used in the analysis allowed us to lack control over the variables of interest to include in the analysis.

## Conclusions

Our study concludes that the trends of receiving TT immunization and adequate doses have decreased over the years in Bangladesh. In addition, the findings suggest that the socio-demographic, socio-economic, and maternal health-related factors are accountable for TT immunization. The official ANC visit, early ANC visit, and exemption of user fees should be considered. Interventions to reduce the observed inequalities between districts, especially the peripheral region, need to be emphasized. Likewise, public health interventions targeting rural women who are uneducated, living in lower wealth quintiles, and women who do not attend ANC facilities can be encouraged for TT immunization. The governments and other stakeholders should raise awareness in promoting female education to correct women’s perceptions of the importance of vaccination across different regions of the country.

## Supporting information

S1 Table Spatial distribution of change rate (MAP). (DOCX)

S2 Table Determinants of tetanus toxoid vaccination (AOR). (DOCX)

S3 Table Determinants of tetanus toxoid vaccination (COR). (DOCX)

S4 Table Determinants of taking adequate doses of tetanus toxoid vaccine (COR). (DOCX)
