# Supplementary material for: Trends and determinants of taking tetanus toxoid vaccine among women during last pregnancy in Bangladesh: Country representative survey from 2006 to 2019
Source: PLoS One. 2022 Oct 20;17(10):e0276417. doi: 10.1371/journal.pone.0276417 (PMC9584373; doi:10.1371/journal.pone.0276417)
Supplement: S1 Table — (DOCX) [file pone.0276417.s001.docx]

S1 Table: Spatial distribution of change rate of tetanus toxoid vaccination status from 2006 to 2019

|  | **Took any TT** | | | | | **Adequate doses of TT** | | | | |
| --- | --- | --- | --- | --- | --- | --- | --- | --- | --- | --- |
|  | **2006** | **%** | **2019** | **%** | **% Of change** | **2006** | **%** | **2019** | **%** | **% Of change** |
| Bagerhat | 71 | 74.7 | 43 | 46.2 | -38.1 | 46 | 64.8 | 22 | 52.4 | -19.2 |
| Bandarban | 29 | 78.4 | 9 | 18.8 | -76.1 | 23 | 76.7 | 2 | 22.2 | -71.0 |
| Barguna | 84 | 93.3 | 39 | 66.1 | -29.2 | 69 | 82.1 | 26 | 66.7 | -18.8 |
| Barisal | 182 | 90.1 | 53 | 50.5 | -44.0 | 139 | 76.4 | 28 | 52.8 | -30.8 |
| Bhola | 135 | 78.0 | 97 | 72.9 | -6.5 | 86 | 63.7 | 53 | 54.6 | -14.2 |
| Bogra | 191 | 80.3 | 87 | 43.3 | -46.1 | 156 | 81.7 | 33 | 37.9 | -53.6 |
| Brahmanbaria | 235 | 79.7 | 124 | 56.6 | -28.9 | 151 | 64.3 | 49 | 39.5 | -38.5 |
| Chandpur | 206 | 92.4 | 43 | 37.1 | -59.9 | 169 | 82.0 | 27 | 62.8 | -23.5 |
| Chittagong | 584 | 89.6 | 299 | 58.7 | -34.4 | 423 | 72.4 | 134 | 44.8 | -38.1 |
| Chuadanga | 64 | 85.3 | 32 | 47.1 | -44.9 | 47 | 73.4 | 17 | 53.1 | -27.7 |
| Comilla | 417 | 91.4 | 235 | 63.9 | -30.2 | 256 | 61.4 | 150 | 63.8 | 4.0 |
| Cox's Bazar | 180 | 80.4 | 87 | 45.8 | -43.0 | 120 | 66.3 | 25 | 28.4 | -57.1 |
| Dhaka | 547 | 83.0 | 332 | 51.4 | -38.1 | 376 | 68.7 | 201 | 60.5 | -11.9 |
| Dinajpur | 176 | 81.9 | 49 | 32.7 | -60.1 | 93 | 52.8 | 13 | 27.1 | -48.7 |
| Faridpur | 126 | 85.1 | 39 | 37.1 | -56.4 | 86 | 68.3 | 23 | 59.0 | -13.6 |
| Feni | 79 | 84.9 | 52 | 52.0 | -38.8 | 51 | 63.8 | 43 | 81.1 | 27.3 |
| Gaibandha | 153 | 86.9 | 93 | 58.1 | -33.1 | 107 | 69.5 | 36 | 38.7 | -44.3 |
| Gazipur | 137 | 82.5 | 116 | 56.9 | -31.1 | 77 | 56.6 | 51 | 44.3 | -21.7 |
| Gopalganj | 72 | 70.6 | 33 | 42.9 | -39.3 | 49 | 68.1 | 24 | 72.7 | 6.9 |
| Habiganj | 167 | 76.6 | 41 | 27.7 | -63.8 | 116 | 69.5 | 14 | 34.1 | -50.8 |
| Joypurhat | 50 | 82.0 | 17 | 43.6 | -46.8 | 34 | 68.0 | 6 | 35.3 | -48.1 |
| Jamalpur | 183 | 81.3 | 59 | 47.6 | -41.5 | 135 | 73.8 | 33 | 55.9 | -24.2 |
| Jessore | 158 | 76.0 | 67 | 41.6 | -45.2 | 77 | 48.7 | 23 | 34.3 | -29.6 |
| Jhalokati | 47 | 87.0 | 25 | 53.2 | -38.9 | 30 | 63.8 | 11 | 44.0 | -31.1 |
| Jhenaidah | 99 | 85.3 | 74 | 62.7 | -26.5 | 51 | 51.5 | 19 | 25.7 | -50.2 |
| Khagrachhari | 57 | 68.7 | 24 | 49.0 | -28.7 | 40 | 70.2 | 23 | 95.8 | 36.6 |
| Khulna | 138 | 78.0 | 50 | 39.4 | -49.5 | 76 | 55.1 | 22 | 44.0 | -20.1 |
| Kishorganj | 254 | 80.6 | 108 | 46.8 | -42.0 | 175 | 68.9 | 52 | 47.7 | -30.8 |
| Kurigram | 156 | 86.2 | 75 | 54.7 | -36.5 | 131 | 84.0 | 55 | 73.3 | -12.7 |
| Kushtia | 100 | 76.3 | 59 | 54.6 | -28.4 | 47 | 47.0 | 15 | 25.4 | -45.9 |
| Lakshmipur | 131 | 87.3 | 71 | 62.3 | -28.7 | 102 | 77.9 | 42 | 59.2 | -24.0 |
| Lalmonirhat | 99 | 81.1 | 60 | 65.9 | -18.7 | 73 | 73.7 | 30 | 50.0 | -32.2 |
| Madaripur | 102 | 87.2 | 25 | 37.9 | -56.6 | 64 | 62.7 | 12 | 46.2 | -26.4 |
| Magura | 69 | 85.2 | 27 | 39.1 | -54.1 | 41 | 59.4 | 11 | 40.7 | -31.4 |
| Manikganj | 84 | 75.7 | 38 | 48.7 | -35.6 | 59 | 71.1 | 19 | 50.0 | -29.7 |
| Meherpur | 44 | 89.8 | 24 | 61.5 | -31.5 | 32 | 72.7 | 11 | 44.0 | -39.5 |
| Maulvibazar | 151 | 74.4 | 29 | 23.0 | -69.1 | 101 | 66.9 | 17 | 58.6 | -12.4 |
| Munshiganj | 89 | 80.2 | 22 | 26.5 | -66.9 | 48 | 53.9 | 15 | 71.4 | 32.4 |
| Mymensingh | 377 | 84.5 | 224 | 65.5 | -22.5 | 264 | 70.0 | 107 | 47.8 | -31.8 |
| Naogaon | 133 | 68.6 | 55 | 50.5 | -26.4 | 82 | 61.7 | 17 | 30.9 | -49.9 |
| Narail | 54 | 74.0 | 16 | 36.4 | -50.8 | 35 | 64.8 | 6 | 37.5 | -42.1 |
| Narayanganj | 179 | 79.2 | 80 | 43.2 | -45.4 | 116 | 65.2 | 34 | 42.5 | -34.8 |
| Narsingdi | 189 | 80.8 | 59 | 48.0 | -40.6 | 124 | 65.6 | 27 | 45.8 | -30.2 |
| Natore | 100 | 82.6 | 46 | 46.9 | -43.2 | 61 | 61.0 | 11 | 23.9 | -60.8 |
| Nawabganj | 99 | 69.7 | 39 | 34.5 | -50.5 | 59 | 59.0 | 15 | 39.5 | -33.1 |
| Netrakona | 177 | 76.0 | 76 | 48.1 | -36.7 | 97 | 54.8 | 32 | 42.1 | -23.2 |
| Nilphamari | 138 | 83.6 | 55 | 50.0 | -40.2 | 91 | 65.5 | 15 | 27.3 | -58.3 |
| Noakhali | 193 | 74.5 | 171 | 75.3 | 1.1 | 136 | 70.8 | 145 | 84.8 | 19.7 |
| Pabna | 166 | 86.5 | 104 | 63.4 | -26.7 | 112 | 67.5 | 29 | 27.9 | -58.7 |
| Panchagarh | 67 | 80.7 | 19 | 27.1 | -66.4 | 36 | 53.7 | 5 | 26.3 | -51.0 |
| Patuakhali | 127 | 87.6 | 69 | 69.0 | -21.2 | 99 | 78.6 | 48 | 69.6 | -11.5 |
| Pirojpur | 60 | 87.0 | 40 | 62.5 | -28.1 | 41 | 68.3 | 25 | 61.0 | -10.8 |
| Rajshahi | 133 | 78.7 | 75 | 45.7 | -41.9 | 72 | 53.7 | 31 | 41.9 | -22.0 |
| Rajbari | 76 | 88.4 | 31 | 49.2 | -44.3 | 59 | 77.6 | 23 | 74.2 | -4.4 |
| Rangamati | 49 | 76.6 | 5 | 11.6 | -84.8 | 44 | 89.8 | 5 | 100.0 | 11.4 |
| Rangpur | 228 | 87.4 | 78 | 42.9 | -50.9 | 163 | 71.5 | 43 | 55.1 | -22.9 |
| Shariatpur | 59 | 63.4 | 37 | 48.7 | -23.3 | 40 | 67.8 | 22 | 59.5 | -12.3 |
| Satkhira | 120 | 90.2 | 64 | 64.0 | -29.1 | 77 | 64.2 | 26 | 41.3 | -35.7 |
| Sirajganj | 238 | 80.4 | 126 | 69.6 | -13.4 | 159 | 66.8 | 60 | 47.6 | -28.7 |
| Sherpur | 111 | 86.0 | 31 | 39.7 | -53.8 | 82 | 73.9 | 21 | 65.6 | -11.2 |
| Sunamganj | 211 | 77.6 | 87 | 37.8 | -51.2 | 133 | 63.0 | 47 | 54.0 | -14.3 |
| Sylhet | 242 | 74.2 | 61 | 23.3 | -68.6 | 158 | 65.0 | 36 | 59.0 | -9.2 |
| Tangail | 211 | 78.4 | 75 | 29.2 | -62.8 | 149 | 70.6 | 29 | 38.7 | -45.2 |
| Thakurgaon | 85 | 85.9 | 26 | 27.4 | -68.1 | 48 | 56.5 | 4 | 16.0 | -71.7 |
