# Supplementary material for: Trends and determinants of taking tetanus toxoid vaccine among women during last pregnancy in Bangladesh: Country representative survey from 2006 to 2019
Source: PLoS One. 2022 Oct 20;17(10):e0276417. doi: 10.1371/journal.pone.0276417 (PMC9584373; doi:10.1371/journal.pone.0276417)
Supplement: S2 Table — (DOCX) [file pone.0276417.s002.docx]

**S2 Table:** Determinants of taking tetanus toxoid vaccination in Bangladesh (2006-2019)

| Variables & Categories | Took tetanus toxoid injection during last pregnancy | | | Took adequate doses of tetanus toxoid during last pregnancy | | |
| --- | --- | --- | --- | --- | --- | --- |
|  | 2006 | 2012-13 | 2019 | 2006 | 2012-13 | 2019 |
|  | AOR [LL - UL] | AOR [LL - UL] | AOR [LL - UL] | AOR [LL - UL] | AOR [LL - UL] | AOR [LL - UL] |
| **Age Ref. (45-49)** | | | | | | |
| 15-19 | 1.92 [0.91 - 4.05] | 3.13 [1.55 - 6.34] ** | 2.59 [1.31 - 5.14] ** | 1.00 [0.38 - 2.59] | 0.90 [0.27 - 3.01] | 0.87 [0.30 - 2.51] |
| 20-24 | 1.53[0.73 - 3.21] | 1.95 [0.97 - 3.89] | 1.27 [0.64 - 2.49] | 0.64 [0.25 - 1.65] | 1.07 [0.32 - 3.52] | 0.71 [0.25 - 2.03] |
| 25-29 | 1.30 [0.62 - 2.71] | 1.81 [0.91 - 3.61] | 0.91 [0.46 - 1.78] | 0.48 [0.18 - 1.23] | 0.78 [0.24 - 2.57] | 0.53 [0.18 - 1.51] |
| 30-34 | 0.90 [0.43 - 1.89] | 1.63 [0.81 - 3.28] | 0.81 [0.41 - 1.60] | 0.42 [0.16 - 1.09] | 0.57 [0.17 - 1.89] | 0.50 [0.17 - 1.45] |
| 35-39 | 0.80 [0.38 - 1.69] | 1.82 [0.90 - 3.69] | 0.93 [0.47 - 1.84] | 0.46 [0.18 - 1.20] | 0.66 [0.20 - 2.22] | 0.40 [0.14 - 1.17] |
| 40-44 | 0.74 [0.33 - 1.68] | 1.62 [0.77 - 3.43] | 0.77 [0.36 - 1.64] | 0.48 [0.17 - 1.36] | 0.59 [0.17 - 2.09] | 0.37 [0.11 - 1.21] |
| **Education Ref. (Never attend school)** | | | | | | |
| Primary incomplete | 0.99 [0.86 - 1.15] | 1.19 [1.00 - 1.43] * | 0.72 [0.23 - 2.31] | 1.01 [0.87 - 1.16] | 0.90 [0.71 - 1.14] | 0.27 [0.05 - 1.57] |
| Primary completed | 1.09 [0.92 - 1.29] | 1.01 [0.85 - 1.20] | 1.03 [0.86 - 1.23] | 0.87 [0.75 - 1.01] | 0.88 [0.70 - 1.11] | 0.85 [0.65 - 1.09] |
| Secondary incomplete | 1.20 [1.03 - 1.40]* | 0.97 [0.83 - 1.13] | 1.04 [0.87 - 1.25] | 0.80 [0.70 - 0.91]** | 0.77 [0.63 - 0.95]* | 0.79 [0.61 - 1.02] |
| Secondary completed or higher | 1.08 [0.86 - 1.37] | 0.93 [0.76 - 1.15] | 0.79 [0.66 - 0.95] * | 0.86 [0.72 - 1.04] | 0.57 [0.44 - 0.74]*** | 0.70 [0.53 - 0.91]** |
| Non-standard curriculum^a^ | 1.35 [0.53 - 3.49] | - | - | 1.56 [0.67 - 3.63] | - | - |
| **Area Ref. (Rural)** | | | | | | |
| Urban | 0.98 [0.86 - 1.12] | 1.17 [1.03 - 1.34] * | 0.82 [0.72 - 0.92] ** | 0.97 [0.86 - 1.08] | 1.04 [0.88 - 1.23] | 1.31 [1.10 - 1.55]** |
| Tribal^b^ | 0.58 [0.36 - 0.93] * | - | - | 1.46 [0.85 - 2.51] | - | - |
| **Wealth index quintiles Ref. (Richest)** | | | | | | |
| Poorest | 0.97 [0.78 - 1.20] | 0.96 [0.79 - 1.17] | 0.99 [0.83 - 1.18] | 0.96 [0.80 - 1.15] | 0.65 [0.50 - 0.83] ** | 1.04 [0.81 - 1.32] |
| Second | 0.98 [0.79 - 1.22] | 1.02 [0.84 - 1.23] | 0.92 [0.78 - 1.09] | 0.97 [0.81 - 1.16] | 0.60 [0.47 - 0.76] *** | 0.98 [0.77 - 1.24] |
| Middle | 0.86 [0.69 - 1.06] | 0.92 [0.77 - 1.10] | 0.90 [0.77 - 1.05] | 0.98 [0.82 - 1.16] | 0.69 [0.55 - 0.87] ** | 1.17 [0.94 - 1.45] |
| Fourth | 0.88 [0.72 - 1.07] | 1.03 [0.87 - 1.22] | 0.89 [0.77 - 1.03] | 0.82 [0.70 - 0.95]* | 0.67 [0.54 - 0.84] *** | 1.19 [0.97 - 1.46] |
| **Division Ref. (Sylhet)** | | | | | | |
| Barisal | 2.24 [1.70 - 2.95] *** | 3.88 [2.96 - 5.08] *** | 4.06 [3.16 - 5.23] *** | 1.32 [1.05 - 1.68]* | 1.89 [1.31 - 2.73]** | 1.38 [0.97 - 1.98] |
| Chittagong | 1.83 [1.51 - 2.23] *** | 4.01 [3.28 - 4.90] *** | 3.20 [2.65 - 3.87] *** | 1.19 [0.99 - 1.42] | 2.20 [1.64 - 2.97]*** | 1.34 [1.00 - 1.81] |
| Dhaka | 1.33 [1.12 - 1.60] ** | 2.30 [1.90 - 2.78] *** | 1.93 [1.60 - 2.33] *** | 0.97 [0.82 - 1.16] | 1.44 [1.08 - 1.92]* | 1.11 [0.82 - 1.51] |
| Khulna | 0.98 [0.78 - 1.22] | 1.79 [1.42 - 2.25] *** | 2.10 [1.69 - 2.61] *** | 0.61 [0.50 - 0.75]*** | 0.53 [0.38 - 0.74]*** | 0.61 [0.43 - 0.85]** |
| Mymensingh^c^ | - | - | 3.18 [2.53 - 3.98] *** | - | - | 0.87 [0.62 - 1.22] |
| Rajshahi | 1.08 [0.89 - 1.30] | 1.91 [1.53 - 2.39] *** | 2.37 [1.92 - 2.92] *** | 0.90 [0.75 - 1.07] | 1.22 [0.88 - 1.69] | 0.54 [0.39 - 0.75]*** |
| Rangpur^d^ | - | 2.47 [1.98 - 3.10] *** | 1.79 [1.45 - 2.21] *** | - | 1.04 [0.75 - 1.43] | 0.76 [0.55 - 1.07] |
| **Has immunization card Ref. (No)** | | | | | | |
| Yes (card seen) | 5.19 [4.50 - 5.98] *** | 3.28 [2.87 - 3.74] *** | 2.14 [1.91 - 2.39] *** | 0.90 [0.80 - 1.02] | 1.62 [1.36 - 1.92]*** | 1.14 [0.98 - 1.32] |
| Yes (card not seen) | 3.43 [3.06 - 3.84] *** | 2.00 [1.79 - 2.24] *** | 1.47 [1.33 - 1.64] *** | 1.15 [1.03 - 1.29]* | 0.99 [0.85 - 1.16] | 0.91 [0.79 - 1.06] |
| **Place of delivery Ref. (Private service^g^)** | | | | | | |
| Respondent's home | 1.00 [0.80 - 1.25] | 1.17 [1.01 - 1.36] * | 0.97 [0.86 - 1.09] | 0.91 [0.77 - 1.08] | 0.94 [0.78 - 1.14] | 1.18 [1.00 - 1.38]* |
| Government service^e^ | 1.41 [1.06 - 1.88] * | 1.09 [0.92 - 1.31] | 1.02 [0.89 - 1.16] | 1.02 [0.83 - 1.26] | 0.88 [0.71 - 1.10] | 1.11 [0.92 - 1.33] |
| Other^f^ | 1.07 [0.84 - 1.36] | 0.84 [0.67 - 1.06] | 0.91 [0.77 - 1.09] | 1.13 [0.94 - 1.37] | 0.78 [0.57 - 1.07] | 1.37 [1.07 - 1.74]* |
| **Received ANC Ref. (No)** | | | | | | |
| Yes | 1.51 [1.35 - 1.69] *** | 1.35 [1.21 - 1.52] *** | 1.50 [1.32 - 1.70] *** | 0.89 [0.81 - 0.98]* | 0.81 [0.70 - 0.95]** | 0.76 [0.63 - 0.92]** |
| *p<0.05, **p<0.01, ***p<0.001 (p indicates *P*-Value)  a= “Non-standard curriculum” data were not collected in 2012-13 5 and 2019.  b= “Tribal” data were not collected in 2012-13 and 2019.  c= Mymensingh division was established in 2015.  d= Rangpur division was established in 1 July 2010.  e= Government services include “Government hospital, Govt. clinic, Govt. health center and Other public services” | | | | f= “Other” include “Other home, and other values provided by MICS.  g= Private services include “Private clinic, private hospital, private maternity home and other private medical” | | |
