# Supplementary material for: Trends and determinants of taking tetanus toxoid vaccine among women during last pregnancy in Bangladesh: Country representative survey from 2006 to 2019
Source: PLoS One. 2022 Oct 20;17(10):e0276417. doi: 10.1371/journal.pone.0276417 (PMC9584373; doi:10.1371/journal.pone.0276417)
Supplement: S3 Table — (DOCX) [file pone.0276417.s003.docx]

S3 Table. Bivariate analyses of determinants of taking tetanus toxoid vaccine (2006-2019)

|  |  |  | **2006** |  | **2012-13** |  | **2019** |
| --- | --- | --- | --- | --- | --- | --- | --- |
| **Variables** | **Categories** | **P-Value** | **COR [LL - UL]** | **P-Value** | **COR [LL - UL]** | **P-Value** | **COR [LL - UL]** |
| Age | 15-19 | 0.001 | 3.17 [1.57 - 6.39] | <0.001 | 5.06 [2.61 - 9.83] | 0.004 | 2.69 [1.38 - 5.24] |
|  | 20-24 | 0.009 | 2.51 [1.25 - 5.03] | 0.001 | 3.10 [1.62 - 5.95] | 0.485 | 1.26 [0.65 - 2.44] |
|  | 25-29 | 0.065 | 1.92 [0.96 - 3.86] | 0.003 | 2.67 [1.39 - 5.12] | 0.788 | 0.91 [0.47 - 1.77] |
|  | 30-34 | 0.567 | 1.23 [0.61 - 2.47] | 0.012 | 2.32 [1.20 - 4.48] | 0.513 | 0.80 [0.41 - 1.55] |
|  | 35-39 | 0.973 | 1.01 [0.50 - 2.05] | 0.011 | 2.37 [1.22 - 4.63] | 0.740 | 0.89 [0.46 - 1.75] |
|  | 40-44 | 0.498 | 0.77 [0.36 - 1.65] | 0.099 | 1.82 [0.89 - 3.71] | 0.246 | 0.64 [0.31 - 1.35] |
|  | 45-49 | Ref. |  |  |  |  |  |
| Education | Primary incomplete | <0.001 | 1.33 [1.16 - 1.52] | 0.001 | 1.33 [1.13 - 1.57] | 0.876 | 1.09 [0.36 - 3.33] |
|  | Primary completed | <0.001 | 1.60 [1.37 - 1.86] | 0.003 | 1.26 [1.08 - 1.48] | 0.002 | 1.30 [1.10 - 1.53] |
|  | Secondary incomplete | <0.001 | 2.16 [1.91 - 2.45] | <0.001 | 1.45 [1.27 - 1.65] | <0.001 | 1.58 [1.34 - 1.85] |
|  | Secondary completed or higher | <0.001 | 2.12 [1.77 - 2.54] | <0.001 | 1.54 [1.31 - 1.81] | 0.003 | 1.26 [1.08 - 1.47] |
|  | Non-standard curriculum^a^ | 0.173 | 1.88 [0.76 - 4.64] | - | - | - | - |
|  | Never attend school | Ref. |  |  |  |  |  |
| Area | Urban | 0.006 | 1.17 [1.05 - 1.30] | <0.001 | 1.37 [1.22 - 1.53] | 0.039 | 0.90 [0.82 - 0.99] |
|  | Tribal^b^ | 0.019 | 0.59 [0.38 - 0.92] | - | - | - | - |
|  | Rural | Ref. |  |  |  |  |  |
| Wealth index quintiles | Poorest | <0.001 | 0.56 [0.48 - 0.66] | <0.001 | 0.71 [0.62 - 0.82] | 0.302 | 1.07 [0.94 - 1.21] |
|  | Second | <0.001 | 0.67 [0.57 - 0.79] | 0.010 | 0.83 [0.71 - 0.96] | 0.290 | 1.07 [0.94 - 1.22] |
|  | Middle | <0.001 | 0.69 [0.59 - 0.82] | 0.003 | 0.80 [0.69 - 0.93] | 0.299 | 1.07 [0.94 - 1.22] |
|  | Fourth | 0.015 | 0.81 [0.68 - 0.96] | 0.216 | 0.91 [0.78 - 1.06] | 0.955 | 1.00 [0.88 - 1.14] |
|  | Richest | Ref. |  |  |  |  |  |
| Division | Barisal | <0.001 | 2.09 [1.62 - 2.70] | <0.001 | 3.88 [3.00 - 5.02] | <0.001 | 4.39 [3.46 - 5.58] |
|  | Chittagong | <0.001 | 1.85 [1.55 - 2.22] | <0.001 | 3.72 [3.07 - 4.50] | <0.001 | 3.26 [2.72 - 3.90] |
|  | Dhaka | <0.001 | 1.37 [1.17 - 1.62] | <0.001 | 2.39 [2.00 - 2.87] | <0.001 | 2.08 [1.74 - 2.49] |
|  | Khulna | 0.007 | 1.33 [1.08 - 1.63] | <0.001 | 2.16 [1.74 - 2.69] | <0.001 | 2.42 [1.98 - 2.97] |
|  | Mymensingh^c^ |  |  |  |  | <0.001 | 3.13 [2.52 - 3.89] |
|  | Rajshahi | <0.001 | 1.42 [1.19 - 1.69] | <0.001 | 2.36 [1.91 - 2.92] | <0.001 | 2.65 [2.17 - 3.22] |
|  | Rangpur^d^ |  |  | <0.001 | 2.84 [2.29 - 3.51] | <0.001 | 2.11 [1.73 - 2.58] |
|  | Sylhet | Ref. |  |  |  |  |  |
| Has immunization card | Yes (card seen) | <0.001 | 5.62 [4.91 - 6.45] | <0.001 | 3.18 [2.81 - 3.60] | <0.001 | 2.27 [2.04 - 2.53] |
|  | Yes (card not seen) | <0.001 | 3.68 [3.30 - 4.11] | <0.001 | 1.91 [1.71 - 2.12] | <0.001 | 1.59 [1.44 - 1.76] |
|  | No | Ref. |  |  |  |  |  |
| Place of delivery | Respondent's home | <0.001 | 0.64 [0.53 - 0.77] | 0.227 | 0.93 [0.82 - 1.05] | 0.043 | 0.91 [0.83 - 1.00] |
|  | Government service^e^ | 0.112 | 1.25 [0.95 - 1.63] | 0.403 | 1.07 [0.91 - 1.27] | 0.788 | 0.98 [0.87 - 1.11] |
|  | Other^f^ | 0.499 | 0.93 [0.75 - 1.15] | 0.002 | 0.72 [0.58 - 0.89] | 0.500 | 1.06 [0.90 - 1.24] |
|  | Private service^g^ | Ref. |  |  |  |  |  |
| Received ANC | Yes | <0.001 | 1.85 [1.69 - 2.04] | <0.001 | 1.49 [1.35 - 1.64] | <0.001 | 1.55 [1.39 - 1.73] |
|  | No | Ref. |  |  |  |  |  |
| p<0.05, **p<0.01, ***p<0.001 (p indicates *P*-Value)  a= “Non-standard curriculum” data were not collected in 2012-13 5 and 2019.  b= “Tribal” data were not collected in 2012-13 and 2019.  c= Mymensingh division was established in 2015.  d= Rangpur division was established in 1 July 2010.  e= Government services include “Government hospital, Govt. clinic, Govt. health center and Other public services” | | | | f= “Other” include “Other home and other values provided by MICS.  g= Private services include “Private clinic, private hospital, private maternity home and other private medical” | | | |
