# Supplementary material for: Trends and determinants of taking tetanus toxoid vaccine among women during last pregnancy in Bangladesh: Country representative survey from 2006 to 2019
Source: PLoS One. 2022 Oct 20;17(10):e0276417. doi: 10.1371/journal.pone.0276417 (PMC9584373; doi:10.1371/journal.pone.0276417)
Supplement: S4 Table — (DOCX) [file pone.0276417.s004.docx]

**S4 Table**. Bivariate analyses of taking adequate doses of tetanus toxoid vaccine (20006-2019)

|  |  |  | **2006** |  | **2012-13** |  | **2019** |
| --- | --- | --- | --- | --- | --- | --- | --- |
| **Variables** | **Categories** | **P-Value** | **COR [LL - UL]** | **P-Value** | **COR [LL - UL]** | **P-Value** | **COR [LL - UL]** |
| Age | 15-19 | 0.736 | 0.85 [0.33 - 2.19] | 0.553 | 0.70 [0.22 - 2.27] | 0.505 | 0.71 [0.26 - 1.95] |
|  | 20-24 | 0.229 | 0.56 [0.22 - 1.44] | 0.809 | 0.87 [0.27 - 2.79] | 0.328 | 0.60 [0.22 - 1.66] |
|  | 25-29 | 0.081 | 0.43 [0.17 - 1.11] | 0.499 | 0.67 [0.21 - 2.15] | 0.125 | 0.45 [0.16 - 1.25] |
|  | 30-34 | 0.050 | 0.39 [0.15 - 1.00] | 0.246 | 0.50 [0.15 - 1.62] | 0.114 | 0.44 [0.16 - 1.22] |
|  | 35-39 | 0.093 | 0.44 [0.17 - 1.15] | 0.378 | 0.59 [0.18 - 1.92] | 0.076 | 0.39 [0.14 - 1.10] |
|  | 40-44 | 0.163 | 0.48 [0.17 - 1.35] | 0.462 | 0.63 [0.18 - 2.18] | 0.124 | 0.40 [0.13 - 1.28] |
|  | 45-49 | Ref. |  |  |  |  |  |
| Education | Primary incomplete | 0.125 | 1.11 [0.97 - 1.27] | 0.486 | 0.92 [0.74 - 1.15] | 0.400 | 0.48 [0.08 - 2.69] |
|  | Primary completed | 0.858 | 1.01 [0.88 - 1.17] | 0.828 | 0.98 [0.79 - 1.21] | 0.706 | 0.95 [0.75 - 1.22] |
|  | Secondary incomplete | 0.413 | 0.96 [0.86 - 1.07] | 0.452 | 0.93 [0.78 - 1.12] | 0.319 | 0.89 [0.70 - 1.12] |
|  | Secondary completed or higher | 0.105 | 0.89 [0.76 - 1.03] | 0.034 | 0.80 [0.65 - 0.98] | 0.028 | 0.77 [0.62 - 0.97] |
|  | Non-standard curriculum^a^ | 0.232 | 1.66 [0.72 - 3.80] | - | - | - | - |
|  | Never attend school | Ref. |  |  |  |  |  |
| Area | Urban | 0.148 | 0.93 [0.85 - 1.03] | 0.001 | 1.27 [1.10 - 1.46] | 0.002 | 1.26 [1.09 - 1.45] |
|  | Tribal^b^ | 0.188 | 1.43 [0.84 - 2.44] | - | - | - | - |
|  | Rural | Ref. |  |  |  |  |  |
| Wealth index quintiles | Poorest | 0.094 | 1.12 [0.98 - 1.28] | 0.002 | 0.75 [0.62 - 0.90] | 0.666 | 1.04 [0.87 - 1.25] |
|  | Second | 0.028 | 1.17 [1.02 - 1.34] | <0.001 | 0.70 [0.58 - 0.85] | 0.145 | 0.87 [0.72 - 1.05] |
|  | Middle | 0.065 | 1.14 [0.99 - 1.31] | 0.006 | 0.76 [0.63 - 0.92] | 0.678 | 1.04 [0.86 - 1.25] |
|  | Fourth | 0.167 | 0.91 [0.79 - 1.04] | <0.001 | 0.71 [0.58 - 0.86] | 0.436 | 1.08 [0.89 - 1.29] |
|  | Richest | Ref. |  |  |  |  |  |
| Division | Barisal | 0.004 | 1.41 [1.12 - 1.77] | 0.001 | 1.87 [1.31 - 2.67] | 0.121 | 1.31 [0.93 - 1.86] |
|  | Chittagong | 0.032 | 1.21 [1.02 - 1.44] | <0.001 | 1.99 [1.49 - 2.66] | 0.152 | 1.24 [0.92 - 1.65] |
|  | Dhaka | 0.439 | 1.07 [0.90 - 1.26] | 0.008 | 1.46 [1.11 - 1.94] | 0.753 | 1.05 [0.78 - 1.41] |
|  | Khulna | 0.001 | 0.71 [0.58 - 0.86] | <0.001 | 0.51 [0.37 - 0.71] | <0.001 | 0.55 [0.40 - 0.76] |
|  | Mymenshing^c^ | - | - | - | - | 0.487 | 0.89 [0.64 - 1.24] |
|  | Rajshahi | 0.709 | 1.03 [0.87 - 1.23] | 0.279 | 1.19 [0.87 - 1.63] | <0.001 | 0.54 [0.39 - 0.74] |
|  | Rangpur^d^ | - | - | 0.880 | 0.98 [0.72 - 1.33] | 0.055 | 0.73 [0.53 - 1.01] |
|  | Sylhet | Ref. |  |  |  |  |  |
| Has immunization card | Yes (card seen) | 0.210 | 0.93 [0.83 - 1.04] | <0.001 | 1.37 [1.16 - 1.60] | 0.258 | 1.09 [0.94 - 1.25] |
|  | Yes (card not seen) | 0.058 | 1.11 [1.00 - 1.24] | 0.072 | 0.87 [0.75 - 1.01] | 0.135 | 0.90 [0.78 - 1.03] |
|  | No | Ref. |  |  |  |  |  |
| Place of delivery | Respondent's home | 0.287 | 1.08 [0.93 - 1.26] | 0.684 | 1.03 [0.88 - 1.21] | <0.001 | 1.29 [1.13 - 1.48] |
|  | Government service^e^ | 0.540 | 1.07 [0.87 - 1.30] | 0.263 | 0.89 [0.72 - 1.09] | 0.153 | 1.14 [0.95 - 1.35] |
|  | Other^f^ | <0.001 | 1.43 [1.20 - 1.69] | 0.063 | 0.76 [0.57 - 1.01] | 0.002 | 1.43 [1.14 - 1.79] |
|  | Private service^g^ | Ref. |  |  |  |  |  |
| Received ANC | Yes | <0.001 | 0.83 [0.76 - 0.91] | 0.006 | 0.83 [0.73 - 0.95] | 0.001 | 0.75 [0.64 - 0.89] |
|  | No | Ref. |  |  |  |  |  |
| p<0.05, **p<0.01, ***p<0.001 (p indicates *P*-Value)  a= “Non-standard curriculum” data were not collected in 2012-13 5 and 2019.  b= “Tribal” data were not collected in 2012-13 and 2019.  c= Mymensingh division was established in 2015.  d= Rangpur division was established in 1 July 2010.  e= Government services include “Government hospital, Govt. clinic, Govt. health center and Other public services” | | | | f= “Other” include “Other home and other values provided by MICS.  g= Private services include “Private clinic, private hospital, private maternity home and other private medical” | | | |
